# *Tmprss6*-ASO as a tool for the treatment of Polycythemia Vera mice

**DOI:** 10.1371/journal.pone.0251995

**Published:** 2021-12-10

**Authors:** Carla Casu, Alison Liu, Gianluca De Rosa, Audrey Low, Aae Suzuki, Sayantani Sinha, Yelena Z. Ginzburg, Charles Abrams, Mariam Aghajan, Shuling Guo, Stefano Rivella

**Affiliations:** 1 Division of Hematology, Department of Pediatrics, The Children’s Hospital of Philadelphia (CHOP), Philadelphia, PA, United States of America; 2 Ionis Pharmaceuticals, Inc., Carlsbad, CA, United States of America; 3 University of Pennsylvania, Perelman School of Medicine, Philadelphia, PA, United States of America; 4 Tisch Cancer Institute, Icahn School of Medicine at Mount Sinai, New York, NY, United States of America; 5 Cell and Molecular Biology affinity group (CAMB), University of Pennsylvania, Philadelphia, PA, United States of America; 6 Raymond G. Perelman Center for Cellular and Molecular Therapeutics-CHOP, Philadelphia, PA, United States of America; 7 Penn Center for Musculoskeletal Disorders, CHOP, Philadelphia, PA, United States of America; Lady Davis Institute for Medical Research, CANADA

## Abstract

Polycythemia Vera (PV) is a chronic myeloproliferative neoplasm resulting from an acquired driver mutation in the *JAK2* gene of hematopoietic stem and progenitor cells resulting in the overproduction of mature erythrocytes and abnormally high hematocrit, in turn leading to thromboembolic complications. Therapeutic phlebotomy is the most common treatment to reduce the hematocrit levels and consequently decrease thromboembolic risk. Here we demonstrate that, by using the iron restrictive properties of the antisense oligonucleotides against *Tmprss6* mRNA, we can increase hepcidin to achieve effects equivalent to therapeutic phlebotomy. We provide evidence that this less invasive approach could represent an additional therapeutic tool for the treatment of PV patients.

## Introduction

Polycythemia Vera (PV) is a myeloproliferative disorder (MPDs) characterized by excessive production of erythroblasts with or without an increase in other myeloid lineage cells. Driver mutations in JAK2 tyrosine kinase [important component of the erythropoietin (EPO) receptor pathway] are central to disease pathophysiology and are present in the vast majority of PV patients. A somatic point mutation (V617F) in exon 14 of the *JAK2* gene is the most common mutation (found in more than 90% of the patients affected by PV) leading to the constitutive activation of the downstream signaling pathways, including the signal transducer and activator of transcription 5 (STAT5), extracellular signal-regulated kinase (ERK), and phosphatidylinositol 3-kinase/AKT [1]. This results in massive overproduction of red blood cells (RBC) and consequent abnormally high hematocrit (HCT) and hemoglobin (Hb) concentration [1–3]. The increased production of RBCs (and possibly other blood cells) leads to increased blood viscosity implicated in the increased risk of venous and arterial thrombosis (i.e. heart attack and stroke) associated with this disease.

Therapeutic phlebotomy, alone or combined with cytoreductive therapy, is ubiquitously used to maintain HCT below 45% to decrease the risk of thrombosis and consequently improve morbidity and mortality [4,5]. In the last decade, the use of ruxolitinib (a JAK1/2 inhibitor) has been approved as second-line therapy for PV patients, but despite its ability to robustly improve PV-associated symptoms, ruxolitinib does not eliminate the driver mutated clone; whether ruxolitinib alters the course of disease progression remains incompletely defined [4,6,7]. Therapeutic phlebotomy, albeit effective in a temporary decrease in HCT, does not produce a disease-modifying effect as HCT is ultimately again increased, requiring additional therapeutic phlebotomy, and most PV patients experience a progressive increase in the allele burden of the mutant JAK2 clone over time. Furthermore, recurrent therapeutic phlebotomy leads to iron deficiency, which is in part beneficial, partially limiting erythropoiesis despite the consequently increased iron deficiency-related symptom burden in PV patients. Recently, the expanding body of knowledge in iron metabolism demonstrates that upregulation of the iron regulatory hormone hepcidin ameliorates abnormal erythropoiesis and prevents or limits iron overload in mouse models of β-thalassemia intermedia and hereditary hemochromatosis [8–12]. We hypothesize that exogenous hepcidin, by further limiting iron availability for erythropoiesis, also enables HCT control in PV. Considering this hypothesis, we previously demonstrated successful HCT control using minihepcidin in a mouse model of PV [11]. Furthermore, a Phase II clinical trial using PTG-300, another hepcidin-mimetic agent, demonstrates HCT control without therapeutic phlebotomy and reverses systemic iron deficiency in PV patients with high therapeutic phlebotomy requirements [13]. Here we further hypothesize that the iron restrictive property of elevated endogenous hepcidin expression using GalNac-conjugated antisense oligonucleotides (ASOs) against *Tmprss6* mRNA (*Tmprss6*-ASO) also normalizes HCT in PV.

## Materials and methods

### Mouse model generation

A mouse model of human PV was generated by crossing a floxed heterozygous *Jak2*^*V617Ffl/+*^ mouse with a mouse expressing Cre recombinase under the control of VAV regulatory element (Vav-iCre^+/-^). Both mouse models were purchased from Jackson Laboratory and maintained and bred in the animal facility at the Children’s Hospital of Philadelphia. *Jak2*^*V617Ffl/+*^ VAV-iCre^+/-^ double transgenic mice developed a PV–like phenotype. The cohort of mice with the PV phenotype was generated by engrafting bone marrow cells from a *Jak2*^*V617Ffl/+*^ VAV-iCre^+/-^ mouse into lethally irradiated Pep Boy B6 Cd45.1 mice (known as Pep Boy). The Pep Boy mice allow us to discriminate between the endogenous cells (which carry the differential *Ptprc*^*a*^ pan leukocyte marker (commonly known as CD45.1)) from the donor cells (which carry the CD45.2 variant) (S1 Fig). Bone marrow from a WT (Wild Type) mouse (C57BL6/j) was also transplanted in Cd45.1 recipient as a reference control. All recipients were 8–10 weeks old female mice. Animals were housed in sterilized cages and with antibiotics (Levofloxacine at a dose of 0.67mg/ml changed once a week) enriched water for two weeks before and after irradiation. Animals received 10 Gy (split dose of 2 × 5 Gy, 4 hours apart) of X-ray irradiation on the day of transplantation (ISOVOLT Titan E Series X-Ray Generators). Bone marrow cells were harvested in sterile conditions and resuspended to the desired concentration in sterile PBS 1X. Each recipient received 3x10^6^ bone marrow donor cells via retro-orbital venous plexus injection one hour after the last dose of irradiation and under anesthesia. Animals’ health was closely monitored to make sure that the drop in weight did not exceed 10% or unexpected death occurred. Animals did not show signs of distress during the time course of the experiment and the rate of mortality was 0%.

### Anesthesia

Isoflurane was administered at a dose of 1–3% using a properly calibrated vaporizer to prep the mice for retro-orbital intravenous injection of bone marrow cells or blood collection. The animals were monitored after each procedure until fully awake.

### Hematological analyzes

The establishment of the phenotype was determined by CBC (Complete Blood Count) analysis 1-month post-transplant. For each mouse, we collected 50μl of blood by retro-orbital puncture under anesthesia three times after transplant. Blood collection occurred at week 0 (1-month post-transplant), three weeks after starting treatment, and at the endpoint of the study (six weeks after starting treatment) (S1, S2A and S2B Tables). Blood analysis was performed at the Translational Core Laboratory (TCL) of the CHOP Research Institute by using an automated Sysmex XT-2000iV analyzer.

### *Tmprss6*-ASO treatment

One month after bone marrow transplant (BMT), CBC analysis showed the establishment of the PV-like phenotype. Animals received GalNac-conjugated antisense oligonucleotides (ASOs) (n = 7) against *Tmprss6* mRNA (*Tmprss6*-ASO) (sequence used: GalNAc Tmprss6 5’GCTTAGAGTACAGCCCACTT 3’) [14] or a non-targeting control ASO (CTRL-ASO) (n = 6) of the same chemistry (sequence used: GalNAc control 5’CCTTCCCTGAAGGTTCCTCC 3’) [14]. Drugs were administered under anesthesia by IP injection starting one day after the first CBC measurement (dose of 5mg/kg), twice weekly for three weeks followed by weekly IP injections for three weeks, for a total of six weeks. All the animals were sacrificed one week after the last injection and blood and organs were collected for further analyzes. At the endpoint, anesthetized animals were euthanized by cervical dislocation.

### Fluorescence-activated cell sorter analysis

To analyze erythropoiesis in the bone marrow (BM) and spleen we stained 1x10^6^ cells per sample with anti-mouse CD71, anti-mouse Ter119, and anti-mouse CD44, markers to study the erythroid compartment. Samples were prepared as s single-cell suspensions and sorted using a FACSCalibur (BD-Biosciences). The results were analyzed with FlowJo software (Tree Star).

### Quantitative RT-PCR

Mouse liver’s total RNA was isolated using RNeasy 96 Kits (Qiagen) according to the manufacturer’s instruction. TaqMan qRT-PCR was performed using One-Step SuperScript qRT-PCR kits (Life Technologies). The sequences of primers/probes used are: mouse *Tmprss6*: forward 5’-ATTCCACGCTGGGCTGTTAT-3’, reverse 5’-CTGGTCAGGCCCCTTCAA-3’, probe 5’-FAM-TGAACCCAGGCCAGGTCCTCCC-TAMRA-3’. Gene expression was normalized to total RNA measured by Quant-iT RiboGreen RNA assay (Molecular Probes).

### Quantification of liver and spleen iron content

Liver and spleen specimens were digested in 1 mL of acid solution (3M HCl, 0.6M trichloroacetic acid) overnight at 65°C after vortexing for 30 minutes (these two steps were performed for two consecutive days). We combined 200μl of chromogen solution (1 volume of 0.1% bathophenanthroline sulfate and 1.4% thioglycolic acid solution, 5 volumes of water, and 5 volumes of saturated sodium acetate) with 10μl of acid extraction. The mixture was incubated at room temperature for 10 minutes and the absorbance was measured at 535nm.

### Serum iron parameters

Serum iron and transferrin saturation were measured by using the Iron/TIBC Reagent Set (BioPacific Diagnostic). Serum hepcidin concentration was measured using Hepcidin-Murine-Compete Elisa kit (Intrinsic LifeSciences, LLC) following the manufacturer’s instructions.

### Statistics

Because the reduction in RBC count and HCT is crucial for the improvement PV phenotype, data on RBC count (and HCT) gathered in the pilot study were used to determine the sample size per group presented in this manuscript (*Tmprss*6-ASO n = 7, CTRL-ASO n = 6). These values represent the optimum number of animals needed to attain statistical significance of P ≤0.05 with a 95% probability. One month post-BMT the animals were randomized to create two groups showing the same average of circulating RBC concentration.

WT post-BMT controls are not included in the statistical analysis and are only used as reference. Outliers were identified using the outlier calculator tool in the prism-GraphPad website. Unpaired 2-tailed Student’s t-test (Mann Whitney test) or 2-way ANOVA (with Tukey’s multiple comparisons tests) were used for the statistical analysis which was performed using Prism 8 software. Results represent mean ± SD. Bars represent standard deviation (SD). Asterisks refer to statistically significant differences: *P ≤ 0.05, **P ≤0.01, ***P ≤0.001, ****P ≤ 0.0001.

### Animal study approval

This animal study was conducted under protocols # IAC 18–001173 approved by the Institutional Animal Care and Use Committee of The Children’s Hospital of Philadelphia.

## Results

Using a standard bone marrow transplant (BMT) approach, we generated a cohort of *Jak2*^*V617Ffl/+*^ knock-in mice, a well-established PV mouse model [3,11,15]. Animals received *Tmprss6*-ASO or control ASO (CTRL-ASO) for six weeks starting one month after BMT, once a robust PV phenotype is established. We used a lower dose of *Tmprss6*-ASO than previously described [9] commensurate with the expected systemic iron levels in this model. We used CTRL-ASO vs *Tmprss6*-ASO in PV mice to determine whether this hepcidin up regulator functions similarly to others to induce a decrease in RBC count and HCT. We use WT mice to determine the robustness of the phenotype in PV mice following BMT. Although it is interesting to speculate whether iron deficiency found in PV patients will translate to a lower dose endogenous hepcidin-inducer requirement relative to diseases of iron overload (e.g. β-thalassemia), no current data to inform this is available.

**Fig 1:**
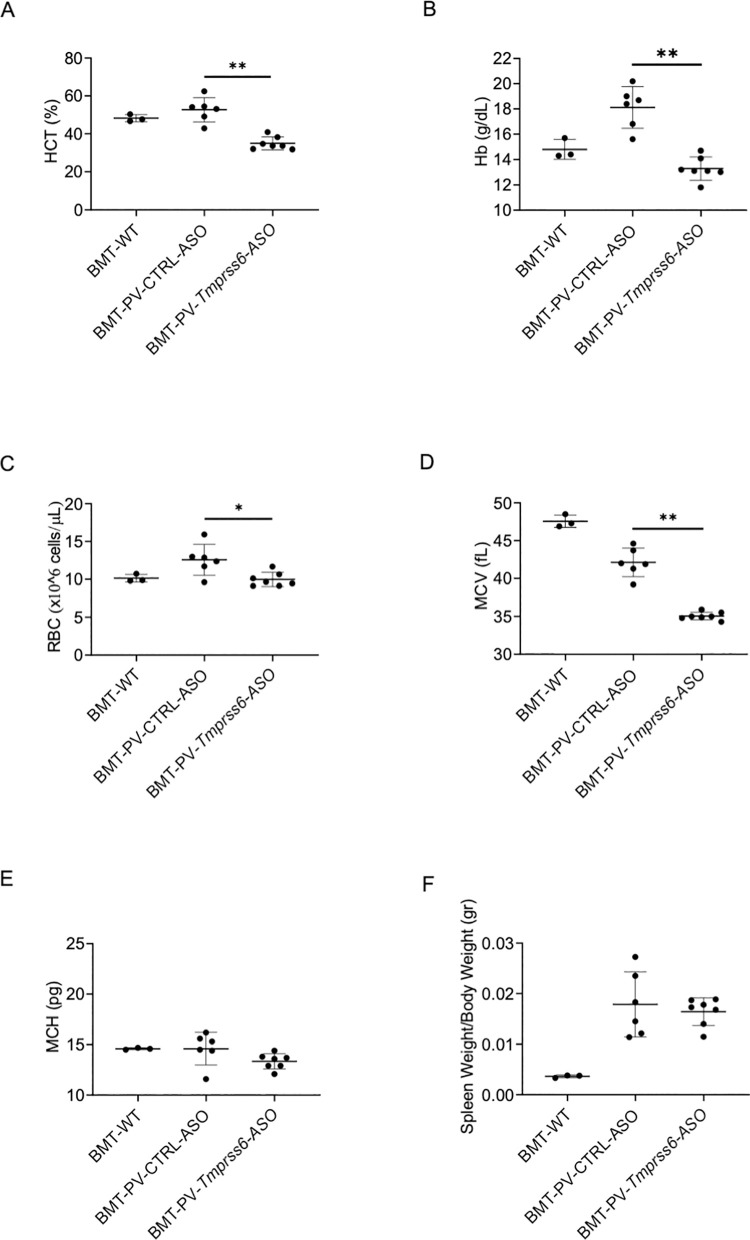
Normalization of the hematological panel of PV animals following *Tmprss6*-ASO treatment. One month after bone marrow transplant (BMT) animals received *Tmprss6*-ASO or CTRL-ASO. Six weeks of *Tmprss6-*ASO administration led to the significant reduction in HCT **(A)** as well as Hb **(B)**, and RBC **(C)** when compared with animals treated with CTRL-ASO and normalized when compared with experimental BMT-WT untreated animals. As expected MCV was significantly reduced **(D)** when compared with CTRL-ASO and BMT-WT animals but no changes were observed in MCH **(E)** and Spleen Size **(F)**. For all the analyzes were used BMT-WT mice (n = 3), BMT-PV-CTRL-ASO mice (n = 6), and BMT-PV-*Tmprss6*-ASO mice (n = 7). Results represent mean ± SD. Bars represent standard deviation (SD). Asterisks refer to statistically significant differences: **P ≤ 0.01, *P ≤ 0.05.

We previously demonstrated that PV mice exhibit very low serum hepcidin concentration at baseline [4]. Furthermore, iron deficiency during expanded erythropoiesis in PV is anticipated based on the enhanced iron requirements necessary to support expanded erythropoiesis. Our results demonstrate that *Tmprss6*-ASO treated animals display a significant increase in serum hepcidin concentration when compared with animals receiving CTRL-ASO (S2A Fig). These findings indicate an effective reduction in *Tmprss6* mRNA expression (S2B Fig) [9]. Overexpression of hepcidin resulted in a dramatic improvement of the phenotype of *Tmprss6*-ASO treated PV mice. Specifically, we demonstrate a significant reduction in the HCT and Hb concentration in *Tmprss6*-ASO treated relative to CTRL-ASO injected mice (Fig 1A and 1B). This is the result of decreased erythrocytosis as indicated by significantly reduced RBC count in *Tmprss6*-ASO treated relative to CTRL-ASO injected mice (Fig 1C). Taken together, *Tmprss6*-ASO leads to normalization of RBC count, Hb, and HCT in PV relative to control untreated mice. As expected, a significant reduction of mean corpuscular volume (MCV) was achieved due to iron restriction but no changes were observed in mean corpuscular hemoglobin (MCH) (Fig 1D and 1E). No change in spleen size was observed in treated animals (Fig 1F). The current results provide an important correlate to the previously published effects of *Tmprss6*-ASO in a mouse model of β-thalassemia intermedia [9]. In this prior study, we demonstrated the iron restrictive effect of *Tmprss6*-ASO in iron overloaded β-thalassemic mice, reversing ineffective erythropoiesis by decreasing spleen erythropoiesis and spleen size (as well as in the bone marrow). In the current work, our data demonstrate that the iron restrictive effect of *Tmprss6*-ASO in PV mice preferentially results in decreased erythropoiesis in the bone marrow. We speculate that a greater effect of *Tmprss6*-ASO on bone marrow erythropoiesis, rather than splenic erythropoiesis, is observed in PV relative to β-thalassemic mice based on the following observations and speculations.

First, we performed an earlier study using a higher dose of *Tmprss6*-ASO in PV mice, demonstrating even more robust iron restriction resulting in anemia and increased splenomegaly (data not shown). The *Tmprss6*-ASO dose used in this study was effective in significantly lowering circulating red blood cell parameters without detrimental effect on splenomegaly. These findings also mimic findings in patients in which PV patients required relatively lower doses of hepcidin-mimetic agents relative to the patients with β-thalassemia, mechanisms of which have not been elucidated. These findings suggest a fundamentally different effect of iron sequestration on erythropoiesis in these diseases. Second, the goal of treatment in PV is to restrict iron availability for erythropoiesis to decrease erythroid differentiation while in β-thalassemia, the goal is to limit the ill-effects of iron overload on erythropoiesis to reverse ineffective erythropoiesis and increase erythroid differentiation to ameliorate anemia. As a consequence, reversal of ineffective erythropoiesis, which predominantly happens in the spleen of β-thalassemic mice, may be more visibly impacted by *Tmprss6*-ASO driven iron restriction in splenic macrophages. Alternatively, iron sequestration in PV mice may increase the “iron restriction response”, leading to decreased erythroid differentiation. This converse effect may account for why the spleen is not decreased in *Tmprss6*-ASO treat PV mice.

Third, while serum erythropoietin is high in β-thalassemic mice, it is reduced or very low in PV mice and patients. This is a central element, denoting that, in this disease, the florid erythropoiesis is independent from EPO-dependent JAK2 activation. Furthermore, *Tmprss6*-ASO leads to a significant decrease in serum erythropoietin in β-thalassemic mice but no change in PV mice (data not shown). This finding suggests that erythropoiesis drive is reduced with decreased erythropoietin in β-thalassemic relative to PV mice, further explaining the differential effect of *Tmprss6*-ASO on erythropoiesis in these diseases.

**Fig 2:**
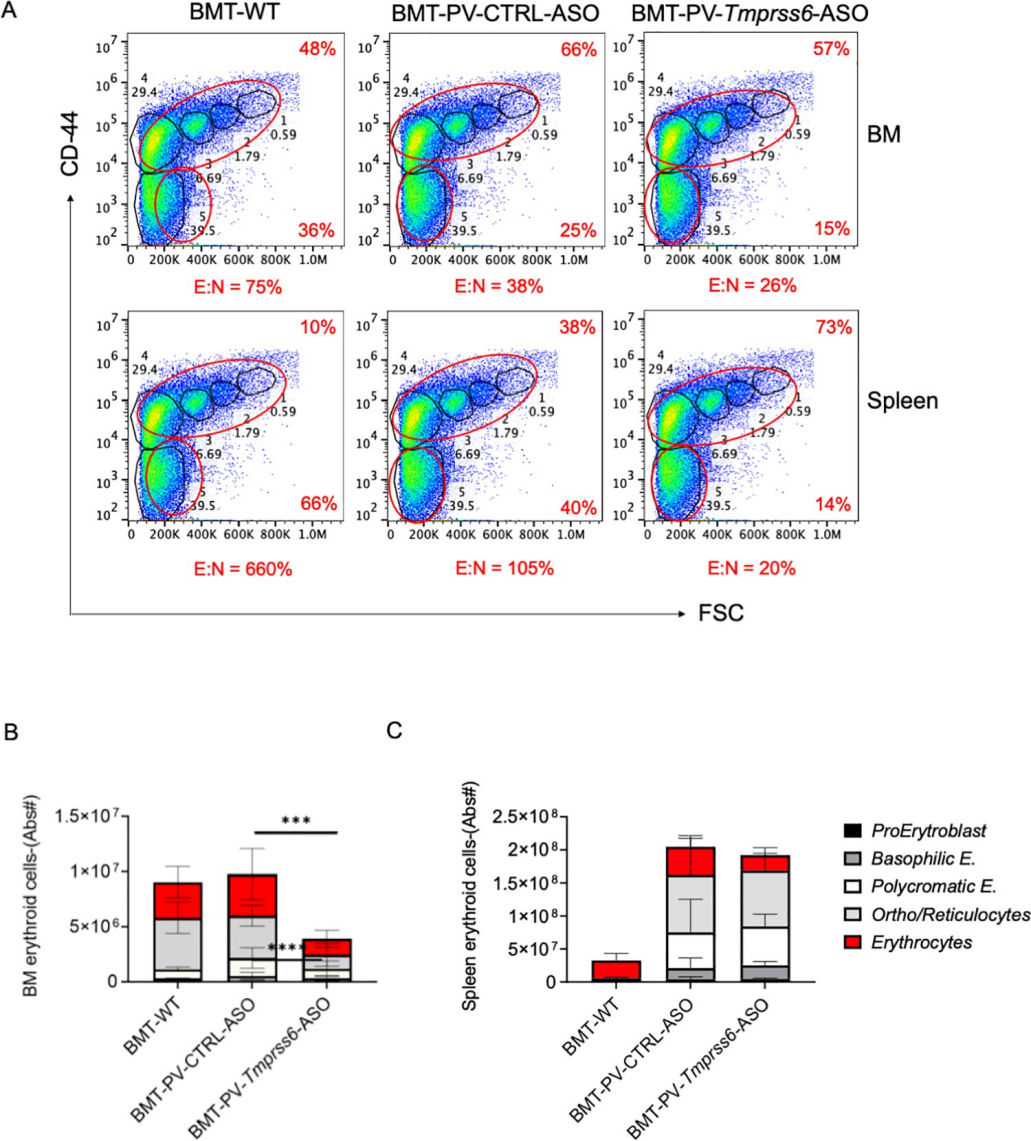
*Tmprss6*-ASO reduced erythropoiesis in the bone marrow of PV mice. Flow cytometry analysis of bone marrow and spleen **(A)** showed signs of iron restricted erythropoiesis as indicated by decreased erythroblast maturation with a decreased ratio of enucleated:nucleated (E:N) cells, consistent with more ineffective erythropoiesis. Flow cytometry analysis of the five clusters of erythroid populations shows significant evidence of decreased erythroblast differentiation in the BM (orthochromatic erythroblasts /reticulocytes, and mature red cells) (B), while only a trend can be appreciated in the spleen (mature red cells) (**C**). For these analyses were used BMT-WT mice (n = 3), BMT-PV-CTRL-ASO mice (n = 6), and BMT-PV-*Tmprss6*-ASO mice (n = 7). Results represent mean ± SD. Bars represent standard deviation (SD). 2-way ANOVA (with Tukey’s multiple comparisons tests) was used for the statistical analysis. Asterisks refer to statistically significant differences: ****P≤ 0.001, ***P≤ 0.005. Only statistical differences between BMT-PV-Ctrl-ASO and BMT-PV-*Tmprss6*-ASO mice are indicated in the graphs.

These results together demonstrate that the iron restrictive effect of *Tmprss6*-ASO requires tailoring based on systemic iron status to derive optimal effects on both hematological parameters and splenomegaly. Iron parameters such as serum iron concentration (S3A Fig) and transferrin saturation (not shown) were not different in *Tmprss6-*ASO treated versus CTRL-ASO injected mice, likely due to less iron utilization when erythropoiesis is reduced in *Tmprss6-*ASO treated mice and despite increased hepcidin levels. As expected, this was associated with an increased iron concentration in the liver (S2B Fig) and spleen (S2C Fig) due to increase retention of iron in macrophages, caused by higher hepcidin concentration. The effect of *Tmprss6-*ASO treatment leads to a clear reduction of RBC in the circulation (Fig 1C) as well as a significant decrease in erythroblast maturation in the bone marrow (BM) (Fig 2A and 2B). These results are in line with our previous studies using a hepcidin agonist [11].

## Conclusions

During the 2020 ASH meeting, data from an ongoing Phase II clinical trial (PTG-300) indicated that a hepcidin mimetic could be effective in controlling HCT without phlebotomy in PV patients [13]. Interestingly, in addition to decreasing RBC count (within 8 weeks of starting treatment), PTG-300 reduces iron deficiency as measured by increased ferritin (within 4 weeks of starting treatment) and MCV (within 16 weeks of starting treatment). Increased ferritin and MCV provide evidence that PTG-300 reverses rather than exacerbates systemic iron deficiency in PV patients. Therefore, compared to preclinical mouse models, the main effect of drugs that increase hepcidin activity in PV patients would be to lower RBC count without exacerbating iron-restricted erythropoiesis. In conclusion, in this study, we provide the proof of principle that *Tmprss*6-ASO could be used in the clinical setting for the treatment of PV patients. Furthermore, an advantage of using *Tmprss6*-ASO is that this kind of compound is administered less frequently (once every 4 weeks, compared to once weekly or more frequently for hepcidin mimetics), with the net benefit of possibly improving compliance while potentially reducing side effects at the injection sites. This is important to enhance available options for treating physicians, but mostly for PV patients who could gain access to a wider range of treatments and, possibly, to improved disease-related outcomes.

## Supporting information

S1 Fig Robust engraftment of CD45.2+ bone marrow cells in lethally irradiated CD45.1+ recipient mice. (TIF)

S2 Fig PV mice treated with *Tmprss6*-ASO display a significant reduction in liver *Tmprss6* mRNA. (TIF)

S3 Fig Increased iron retention in liver and spleen after *Tmprss6*-ASO treatment. (TIF)

S1 Table Hematological parameters at Weeks 0 for BMT-PV-CTRL-ASO and BMT-PV-*Tmprss6*-ASO mice. (TIF)

S2 Table Average of the Hematological parameters at Weeks 0, 3, and 6 for BMT-WT, for BMT-PV-CTRL-ASO, and BMT-PV-*Tmprss6*-ASO mice. (TIF)

S1 Dataset (XLS)
