# Supplementary figures and images for: Tmprss6-ASO as a tool for the treatment of Polycythemia Vera mice
Source: PLoS One. 2021 Dec 10;16(12):e0251995. doi: 10.1371/journal.pone.0251995 (PMC8664179; doi:10.1371/journal.pone.0251995)

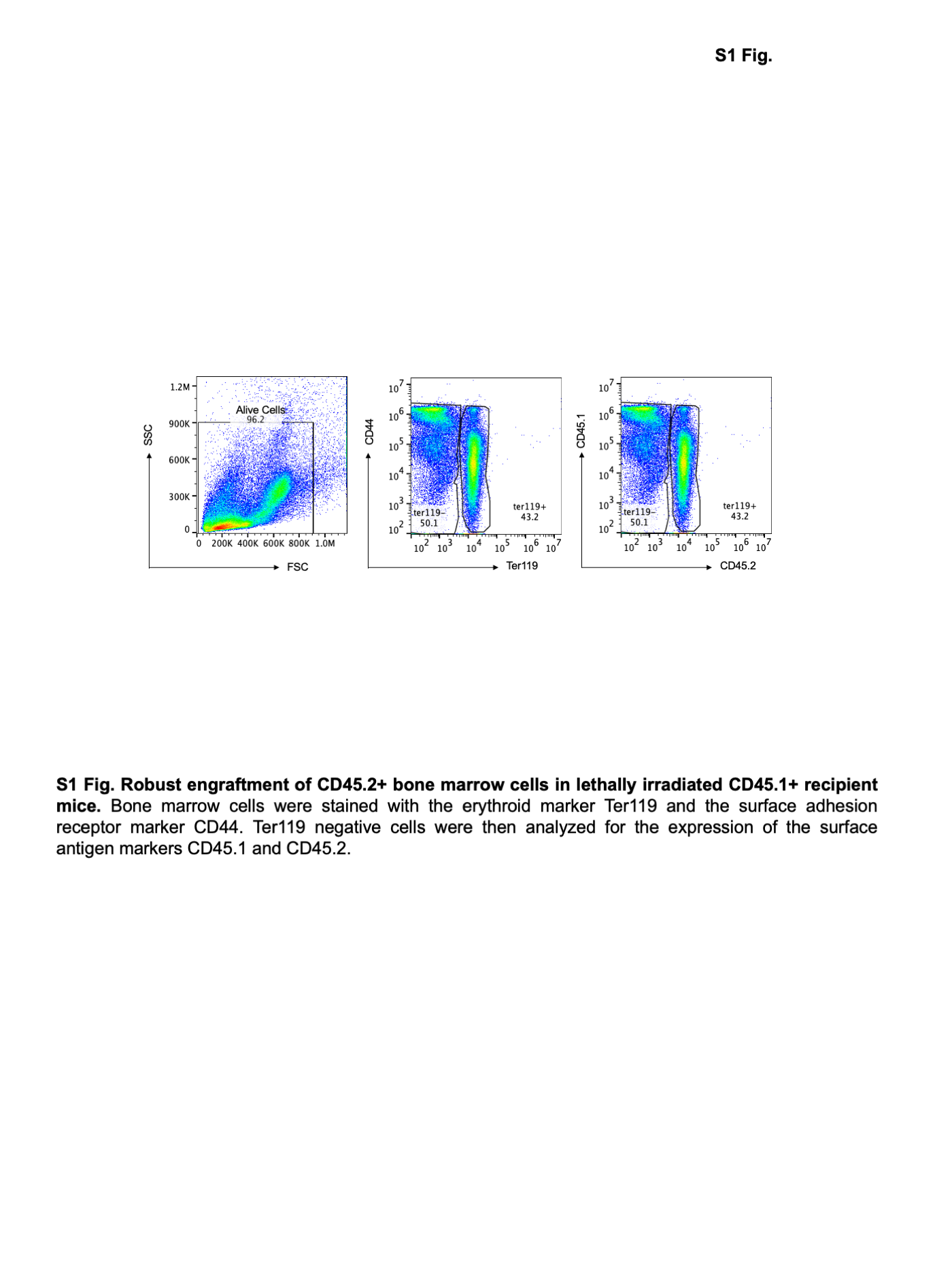

Supplement: S1 Fig — (TIF) [file pone.0251995.s001.tif]

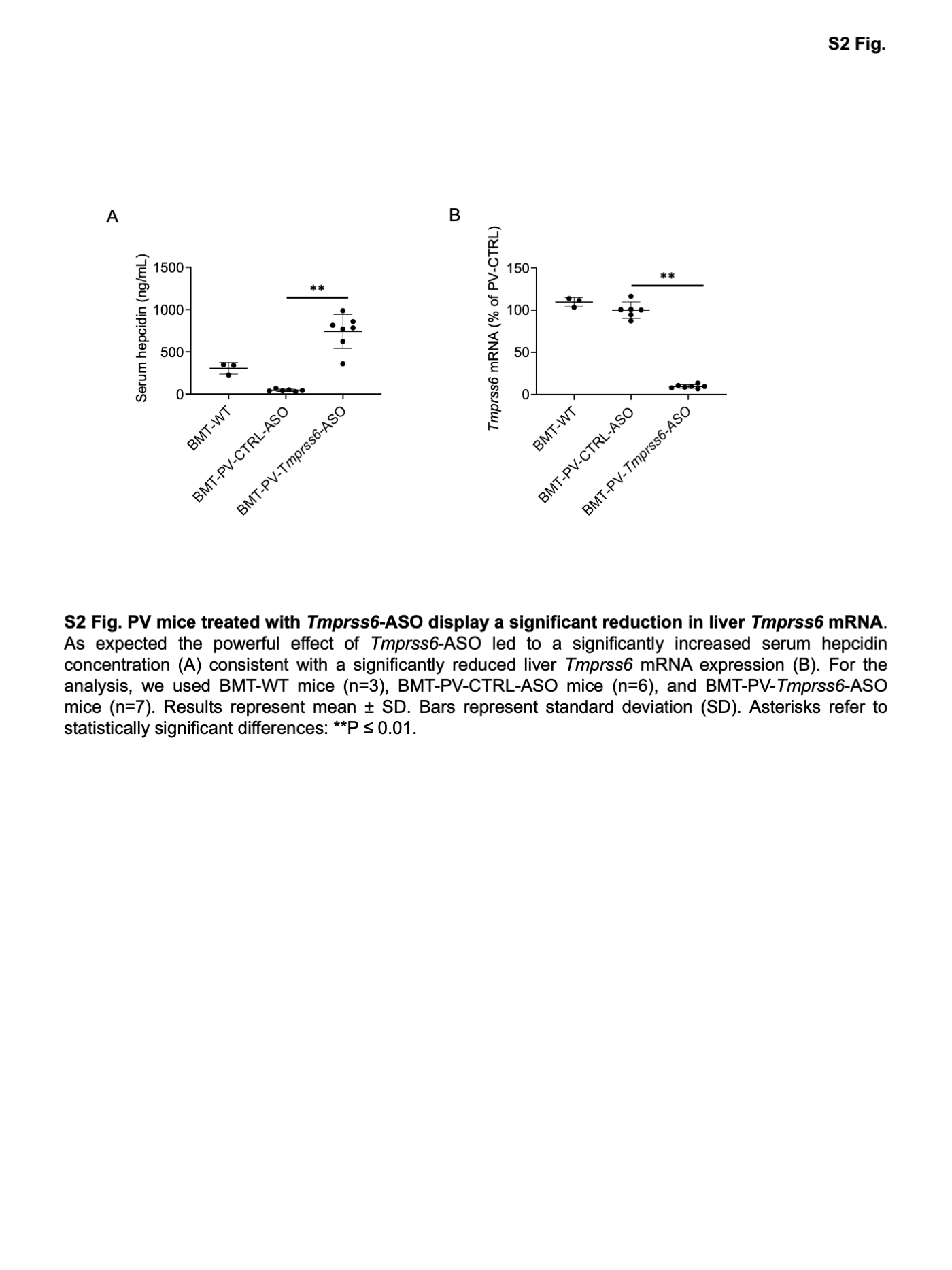

Supplement: S2 Fig — (TIF) [file pone.0251995.s002.tif]

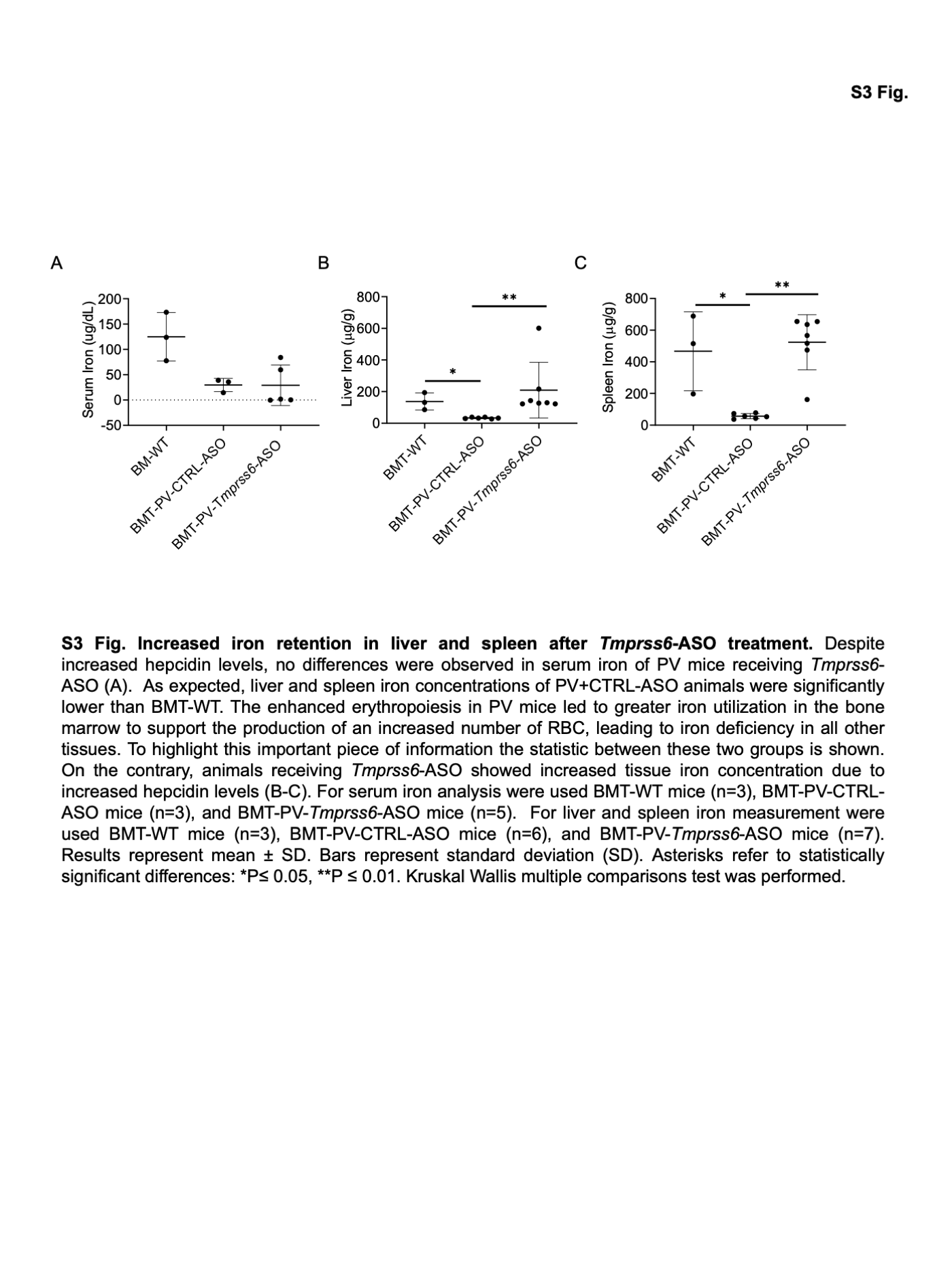

Supplement: S3 Fig — (TIF) [file pone.0251995.s003.tif]

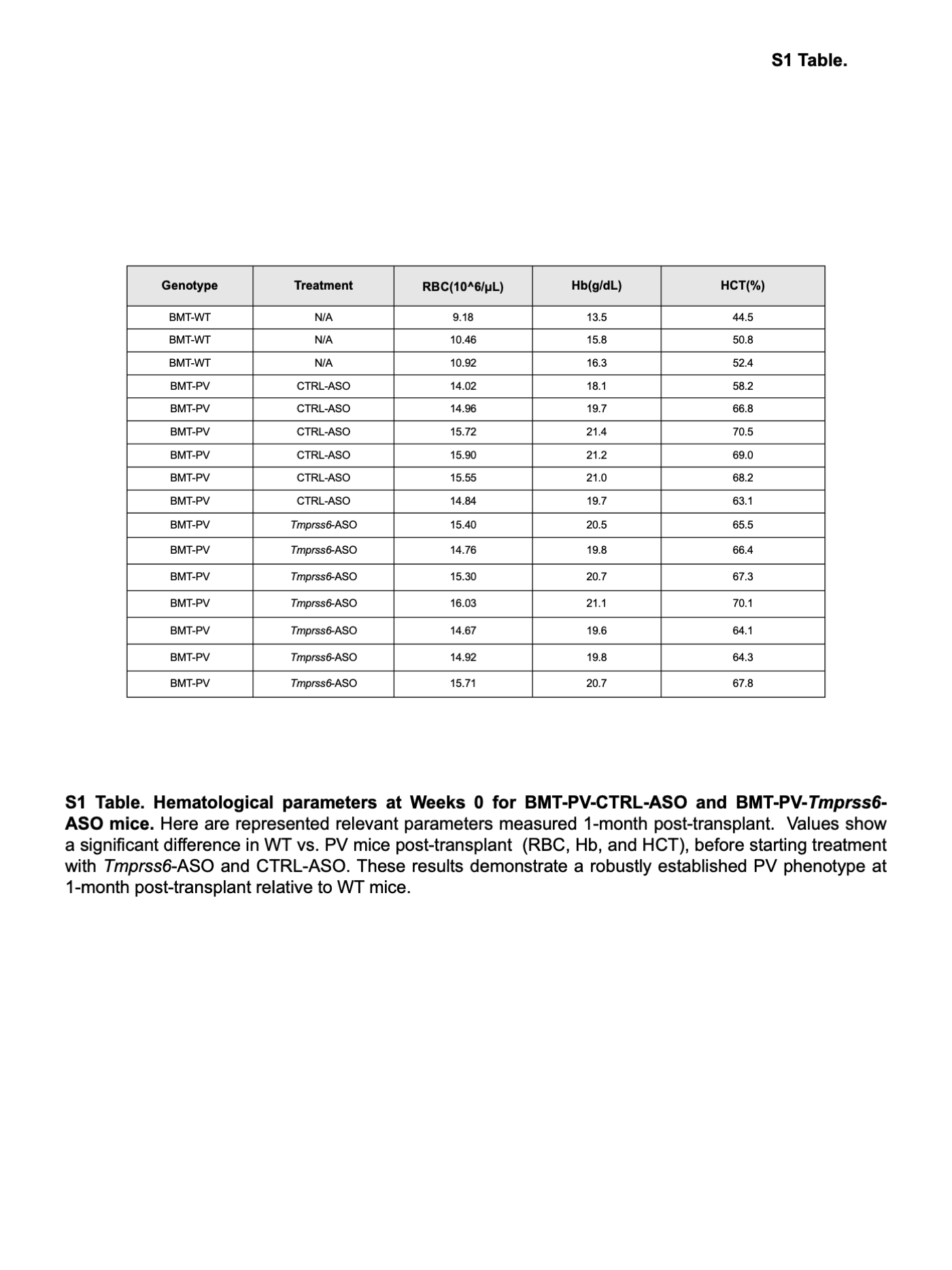

Supplement: S1 Table — (TIF) [file pone.0251995.s004.tif]

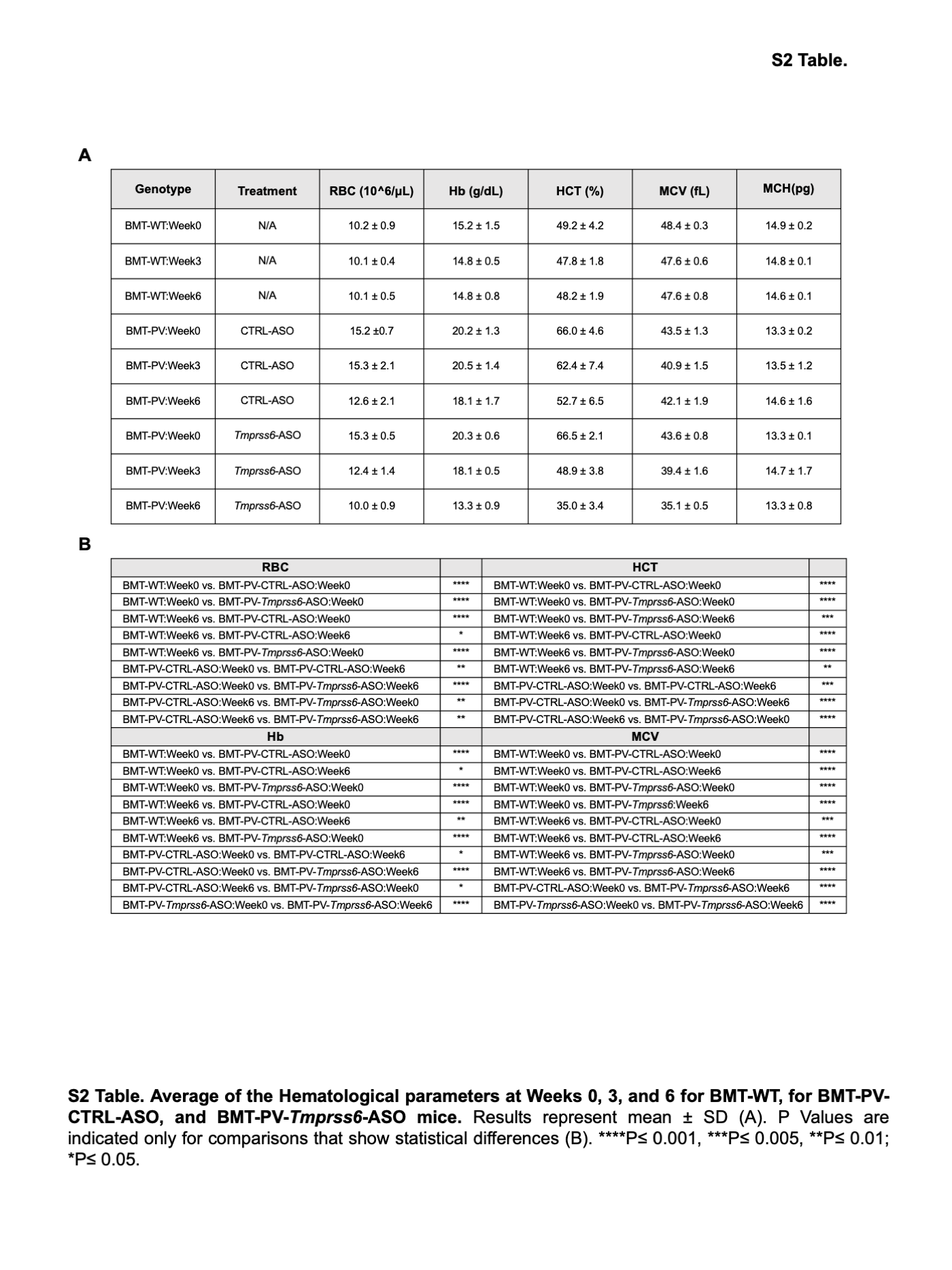

Supplement: S2 Table — (TIF) [file pone.0251995.s005.tif]
